# Participatory eHealth development to support nurses in antimicrobial stewardship

**DOI:** 10.1186/1472-6947-14-45

**Published:** 2014-06-05

**Authors:** Jobke Wentzel, Lex van Velsen, Maarten van Limburg, Nienke de Jong, Joyce Karreman, Ron Hendrix, Julia Elisabeth Wilhelmina Cornelia van Gemert-Pijnen

**Affiliations:** 1 Department of Psychology, Health & Technology, University of Twente, Enschede, the Netherlands; 2 National Institute for Public Health and the Environment (RIVM), Bilthoven, the Netherlands; 3 Department of Technical and Professional Communication, University of Twente, Enschede, the Netherlands; 4 Department of Medical Microbiology, University of Groningen, University Medical Center Groningen, Groningen, the Netherlands

**Keywords:** Participatory development, eHealth, Antimicrobial stewardship, Task support, Nurses

## Abstract

**Background:**

Antimicrobial resistance poses a threat to patient safety worldwide. To stop antimicrobial resistance, Antimicrobial Stewardship Programs (ASPs; programs for optimizing antimicrobial use), need to be implemented. Within these programs, nurses are important actors, as they put antimicrobial treatment into effect. To optimally support nurses in ASPs, they should have access to information that supports them in their preparation, administration and monitoring tasks. In addition, it should help them to detect possible risks or adverse events associated with antimicrobial therapy. In this formative study, we investigate how nurses’ can be supported in ASPs by means of an eHealth intervention that targets their information needs.

**Methods:**

We applied a participatory development approach that involves iterative cycles in which health care workers, mostly nurses, participate. Focus groups, observations, prototype evaluations (via a card sort task and a scenario-based information searching task) and interviews are done with stakeholders (nurses, managers, pharmacist, and microbiologist) on two pulmonary wards of a 1000-bed teaching hospital.

**Results:**

To perform the complex antimicrobial-related tasks well, nurses need to consult various information sources on a myriad of occasions. In addition, the current information infrastructure is unsupportive of ASP-related tasks, mainly because information is not structured to match nurse tasks, is hard to find, out of date, and insufficiently supportive of awareness. Based our findings, we created a concept for a nurse information application. We attuned the application’s functionality, content, and structure to nurse work practice and tasks.

**Conclusions:**

By applying a participatory development approach, we showed that task support is a basic need for nurses. Participatory development proved useful regarding several aspects. First, it allows for combining bottom-up needs (nurses’) and top-down legislations (medical protocols). Second, it enabled us to fragmentise and analyse tasks and to reduce and translate extensive information into task-oriented content. Third, this facilitated a tailored application to support awareness and enhance patient safety. Finally, the involvement of stakeholders created commitment and ownership, and helped to weigh needs from multiple perspectives.

## Background

Antimicrobial resistance (AMR) poses a threat to patient safety worldwide. The prudent use of antimicrobials in Antimicrobial Stewardship Programs (ASPs) is propagated as a means to combat AMR. ASPs aim at optimizing antimicrobial treatment regarding type of antimicrobial, dose, duration, and route of administration. Formulary-based prescribing, expert (microbiologist, infectiologist) consultation, and scheduled re-evaluation of therapy appropriateness lie at the core of ASPs. In addition to often mentioned key-stakeholders in ASPs (e.g., prescribers, clinical microbiologists, and pharmacists), nurses can make an important contribution, even though their role has not yet been fully defined [1-3]. Nurses spend much time with the patient and put antimicrobial therapy into effect: they prepare, administer, and monitor the effects of antimicrobial treatment and possibly related critical events. To ensure optimal antimicrobial treatment, nurses need to pro-actively alert physicians or relevant experts when problems or abnormalities occur. Decision-making in an ASP is complicated by a multitude of experts and information sources, such as medical protocols, formularies, and local guidelines for antimicrobial use. Since different medical professionals cooperate in ASPs, these sources are not always targeted at nurses or created to support their information need. Therefore, in practice, nurses require adequate information support, especially since their task load is already diverse and high.

To enhance their potential role in ASPs, nurses need adequate training and education on relevant antimicrobial topics, or, as Charani et al. [4] put it: “…understanding of the key principles of microbiology and the unwanted consequences of antimicrobial use”. However, at a basic level, information flow and reference sources should be in place and function properly to support nurses’ ASP tasks. Thus, good information support, aimed at nurse tasks in antimicrobial use is a prerequisite for nurse involvement in ASP. Information (such as protocols, instructions, and reference books) should be geared to the practical tasks of preparing and administering antimicrobials. This is equally true for more complex tasks involving other medical specialists, such as monitoring patient change and informing, alerting, or alarming others in case of abnormalities.

Technology or eHealth can be used to support education and information sharing in clinical settings. Various studies describe the development of technologies that can improve information supplies to healthcare workers (HCWs) and/or patients, such as a website with protocol-based information, or the utilization of integrated displays on ICUs [5-7]. Specifically aimed at ASPs, research has shown that eHealth holds potential [8]. To support tasks and match with workflow, the technology should fit the users’ needs as well as the clinical context in which it is implemented. The Centre for eHealth Research and Disease management (CeHRes) provides a “roadmap”: a guideline for the participatory development and implementation of eHealth technology [9]. It offers an approach to systematically anticipate on stakeholders’ (e.g., HCWs, management) needs and values, and to guide design and facilitate implementation. Especially during early stages of development, stakeholder involvement is crucial to create sustainable technologies. A participatory development approach can help to overcome mismatches between work practice and technology. This can be done by checking findings regarding needs, context, and possible design and functionalities with various relevant stakeholders during every stage of development [9].

**Figure 1:**
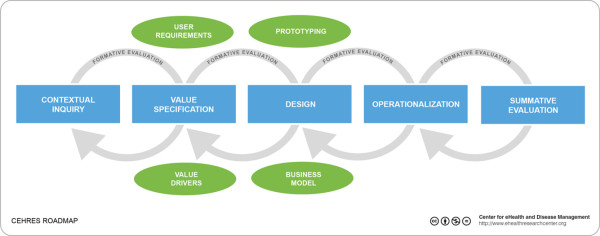
**CeHRes Roadmap.** The CeHRes Roadmap consists of five iterative development cycles: contextual inquiry, value specification, design, operationalization and summative evaluation.

In this study, we applied the CeHRes-Roadmap [9] to perform the formative research phases of contextual inquiry, value specification, and design evaluations (see Figure 1). Contextual inquiry entailed a twofold investigation: Which information problems do nurses encounter when fulfilling which ASP-associated tasks? The outcomes of this stage served as input for the value specification and design. Thus, first we set out to identify the information needs of nurses performing antibiotic-related tasks in a complex environment. Secondly, we investigated how these needs can be met by eHealth technology.

## Methods

### Setting

The pulmonary ward (57 beds) of a Dutch 1000-bed teaching hospital participated in this study. At the time of the study, 62 nurses (45FTE) worked at the pulmonary ward. Although some high-risk wards have their own infection control nurses in this hospital, the pulmonary ward does not. This ward receives support from infection control experts to whom wards were assigned by the infection control department (4.4 FTE).

Key stakeholders involved in the development process, besides the researchers, were nurses (a total of 15, who participated in one or several phases), a nurse manager, a clinical microbiologist, a hospital pharmacist, and in later phases, two programmers. In earlier research [10], these stakeholders were identified as key stakeholders. All participants were informed about the study and signed an informed consent form prior to participating. Ethical approval for this study was not necessary according to dutch law, as participation posed no risk to participants’ physical or emotional integrity. The specific procedures per research method are discussed below.

### Contextual inquiry

Two consecutive ninety-minute-long focus groups were held nurses, a nurse manager, and a pharmacist, to establish what information and information sources are used and needed by nurses to carry out their tasks related to antimicrobial use. We aimed at discussing different situations and at creating an image of nurse working situations and needs that is diverse and as complete as possible. Being able to instantly react to what others say, especially allows for a dynamic discussion that provides the researcher with a broad view on the topic [11].

During the first focus group, conducted by JW and TM (see Acknowledgements), five nurses participated. Four nurses participated during the second focus group, conducted by TM. The nurse manager and pharmacist participated in both focus groups. The nurses had a wide range of work experience, and were reimbursed for their participation with a gift certificate.

Scenarios addressing crucial moments in antimicrobial use were created with a clinical microbiologist and a pharmacist. A scenario was created for each phase in the care process: admitting the patient, preparing/administering antimicrobials, monitoring the effects of treatment, and discharging the patient. An example of a scenario for the admission phase is described in the section below.

### Example scenario in focus group

Mrs. Jansen (55 years old) was referred from the acute care unit to the chest physician. Mrs. Jansen’s temperature is 39 degrees Celsius, she feels clammy and, when listening to her lungs, the physician suspects a lobar pneumonia. Additional diagnostic tests are requested, Mrs. Jansen is admitted to the lung ward, and empirical therapy (Amoxacilline-clavulanate potassium) is started.

During the first focus group, the participants were asked to explain what they would do in each scenario by responding to the questions in the section below. They were asked to write their answers down on post-its, which were then discussed plenary.

### Focus group questions to elicit information needs

1. What information do you need?

2. Where would you get this information?

3. At what moment do you need the information?

4. What problems do you encounter regarding the aforementioned questions?

The transcriptions of the audio recordings that were made during the focus groups were independently analysed by two researchers (JW and TM). A coding scheme was used that was largely based on the focus group set-up, and was complemented with additional themes that emerged from the data, utilizing a pragmatic approach [12]. We searched for expressions indicating information types and the context of use, and possible problems when searching for or using information. Adapted from a taxonomy that resulted from similar research [13], we coded the information types into a) patient-specific (all information that applies to one specific patient only such as prescriptions, medical background, or laboratory results) and b) domain-specific information (information that applies throughout the institute or domain, such as reference book information on a disease, or local or national protocols). Differences in coding by the two independent researchers were discussed until agreement was reached. Hereafter, the results were discussed with a pharmacist to ensure against missing important information.

### Value specification

To create an eHealth application concept that targets the information problems that were uncovered in the contextual inquiry, requirements were elicited and prioritized by the stakeholders.

### Focus group

During the second focus group (see above), participants were shown several examples of how technology can be used to make information available in a task-supporting way. Among others, the website http://www.MRSA-net.nl[5] was shown as an example of a readily available application for protocol communication. Digital patient chart systems and infection control Wikis were discussed as examples of technologies that are not (yet) available in this hospital. The questions, as summed up in the section below, were asked to elicit a discussion on the examples regarding content, type of technology used, and implementation.

### Focus group questions regarding technology needs

1. Is this example useful to you?

2. If so, in what context?

3. If not, why not?

4. What additional information or functionality do you need?

The audio recordings of this part of the focus group were transcribed and translated into design requirements by applying a requirement analysis method [14]. Two independent coders (JW and LvV) analysed those parts of the transcripts that are related to eHealth technology, and translated those into underlying values, attributes of needs and actual technology requirements.

### Observation

To ensure that the requirements are compatible with actual nursing behavior (practice), we validated them via on-site observations. One researcher (JW) accompanied three nurses during moments that are relevant in antimicrobial therapy: morning drug delivery, lunch-time drug delivery, and patient rounds. The observations lasted four hours. The researcher mostly observed quietly, but asked for clarification when needed. She especially looked for crucial moments of (possible) information technology use and preferred device/source, as the requirements were undecided in these areas. Afterwards, the researcher discussed the observations and requirements with the nurse, the nurse manager, and the other stakeholders, to interpret the observations and to resolve remaining conflicts between requirements. This allowed for the creation of a prioritized list of requirements ensuring the best (foreseeable) match with day-to-day nursing tasks. Based on this list, a working prototype was built.

### Design

To assess whether the prototype matches day-to-day practice, a card sorting task, a scenario-based test, and a prototype evaluation were carried out. The assessment was conducted in one-hour individual sessions, with a convenience sample of ten nurses varying in age, work experience, and gender. Each session was guided by one researcher (JW) and audio-recorded. The resulting audio files were summarized.

### Card sorting task

Since the information sources that were identified during the contextual inquiry are created by experts, the structure of the information may not support nurses’ work practice. We validated the information structure of the prototype via a card sorting task. Card sorting is a design method that can be used to create an information structure for a website or application [15]. During the tasks, each participant was given a set of 42 cards with printed parts of protocols, reference documents and other information documents. Participants were asked to sort the cards in (what they regard as) logical groups, and to name each group while thinking aloud. If necessary (e.g., when only two groups were created such as “relevant” and “irrelevant”), the participant was encouraged to further divide the groups into meaningful subgroups. The resulting sorts were analysed using cluster analysis with Websort; an online card sorting program (recently taken over by Optimal Workshop, http://www.optimalworkshop.com). The data were analysed to determine the final information architecture, based on various (iterative) steps [16]: a) percentage of item agreement between the participants (how often two items were placed in one group), b) dendrogram output (visualizations of possible groups, based on item agreement) and c) logical interpretation by the researcher (JW), to make sure no illogical or one-item groups exist due to misinterpretation of cards. Average agreement percentages per final created group were calculated.

### Information searching task

After the card-sorting task, a scenario-based information searching task was conducted to assess whether the prototype fits with current information use and work practice. First, the prototype was introduced briefly. Then, the nurse was given a scenario and asked to resolve it by searching for information, while using the prototype and thinking aloud. The scenarios addressed possible critical moments in correct antimicrobial use such as possible liquid restrictions of patient, drug-drug interactions, correct preparation and concentration, side effects and renal functioning. An example of a scenario is shown in the subsection below.

### Example scenario as used in information searching task

A patient (male, 59 years old) is admitted to the hospital because he is hypoxic, has large infiltrates on his x-thorax, and the rapid test indicates pneumocystis. Since quite some time, the patient takes Prednizon for leukemia. The physician prescribes 1960 miligrams of cotrimoxazol, twice a day. In the past, the patient suffered a deep-veneuse trombose in his leg, for which he is still taking Coumarine. The patient also takes Lasix for cardiomyopathy.

### Prototype evaluation

The session was concluded with a short evaluation of the prototype, consisting of their first impression and comments on the look and feel and content. We also asked for missing features and other thoughts on functionality and implementation in the care process. Regarding the scenario tests and prototype evaluation we merely noted if problems or concrete verbalizations of needs occurred, not how often, as the number of times a problem arises or an idea is expressed may not be representative of its importance [14]. The resulting changes and additions to the requirements were discussed with the other stakeholders (manager, microbiologist, pharmacist, and programmers), to decide on the priority of proposed adjustments for the final version of the application.

## Results

### Contextual inquiry

#### Information need

The focus group results show that nurses need patient-specific information regarding medical history and treatment such as previous admissions, allergies and readily received medications/treatment. Also, practical patient-specific information is needed such as the patient’s identity, the room assigned to a patient, and patient transport arrangements. For example, one of the participants wrote down the following two questions that arose regarding the preparation and administration scenario: *“how often [should we give] antibiotics per day?”* and “*Is Mrs. Jansen allergic to something”*. During the discussion, one nurse explained her information need as written on her post-it: *“Well, first [I need] basic information such as: who this patient is, why the patient is admitted, so, the reason of admittance. What antibiotic is started, how often per day. How many milligrams*” (Nurse 6). For a full list of all patient-related information needs we refer to Additional file 1. Domain-specific information needs regarding the antimicrobial treatment of patients consist of several types of information. For example: *“And what else do you do: the checks, you need to know about the side effects and whether the medication can be given with other medications, a combination”* (Nurse 5). In sum, the following types of information were mentioned:

– background information about a disease that requires antimicrobial treatment or about an antimicrobial (such as characteristics or standard dose);

– instructions on how to dissolve and administer an antimicrobial;

– information on the availability of an antimicrobial (on the ward); and

– information on monitoring such as acute effects, side effects, interactions, and safety of combinations of medicines, possibility of administering several medicines via one intravenous line, and other monitoring points of attention.

Instructions are always used when preparing and administering antimicrobials. However, other domain-specific information needs arise occasionally, especially when caring for patients who are treated with antimicrobials that are not used often on that ward.

### Information sources

Patient-specific and domain-specific information, as described above, are sought from various sources. Some of these sources are used interchangeably, and nurses have indicated that the exact situation, the available search time, and ease of retrieval which source is chosen. Many sources are needed recurrently and are not confined to one single task or phase in the care process. With regard to the scenario of administering antimicrobials, one nurse states: *“Now I would get it [the information] from the electronic prescription system, because that holds my assignment. So where do you get it? From my computer”* (Nurse 1). Patient-specific information is sought in the following paper or electronic sources: the transferal document of the Accident & Emergency unit or admission office, the patient chart, the (electronic) patient record, the general practitioner’s patient record, the electronic prescription system, old admission documents, the digital bed schedule, transfer notes, the assignment sheet, and test results. Domain-specific information is retrieved from protocols in the document management system, the intranet, pharmacological reference books, the hospital’s pharmacy information system, drug instruction leaflets, the electronic prescription system, the assignment sheet, and pharmacy stock lists. For example, a nurse indicates that at the ward they use the drug instruction leaflet for domain specific information: “*You can always find it in the drug instruction leaflet”* (Nurse 2). Besides documents or administration, nurses consult the following persons for patient-specific or domain-specific information: the patient himself/herself, the patient’s family, the secretary, the pharmacist, the physician or resident, the general practitioner, the coordination office for admission and discharge, the physiotherapist, the dietician, or a colleague. A nurse explains regarding asking colleagues: *“Or ask a colleague who does have experience with it, that is also possible of course*” (Nurse 5).

### Work context

Much variation exists regarding the moment in the care process and the context in which information is used. Most information types (such as patient background or status) are used frequently, repeatedly, and throughout the care process, with some exceptions for moment-specific information needs such as instructions for preparing antimicrobials, or information regarding continuation of care at home after discharge.

### Problems with information search

The main problems nurses encounter during information use arise in finding the information, both in terms of locating paper-based resources and concerning searching for information digitally. Nurses report that sometimes, information is not available, for example regarding information on intravenous administration: *“It is located in Pharmacy Net [intranet pharmacy site]. It is not always entirely complete”* (Nurse 3). Information system user-friendliness is also a problem: *“Yes, the document management system. Try to find something in there…”* (Nurse 1). In addition, time constraints hinder information-finding, as illustrated by the following remarks: *“Searching simply costs a lot of time”* (Nurse 3). As a result, nurses may prefer to rely on their own or their colleagues’ knowledge.

The results show that nurses have many instances of patient-specific and domain-specific information needs, and that they turn to a wide variety of sources in order to find the information. Having relevant and usable information at their disposal is important. Yet, the nurses currently lack information sources that are quickly accessible, easy to use, and thus genuinely supportive of their tasks. This non-supportive information infrastructure can stand in the way of nurses taking up, and even wanting to take up an active role in antimicrobial stewardship.

### Value specification

Based on the requirements elicitation procedure, the following values were identified from the focus group results: easy access to information, useful and relevant information, and ease of use of information. This is illustrated by the following quote: *“You need to have it directly at hand whenever you need it, that you can click on it and reach the right page directly (…) that you don’t have to search for too long”* (Nurse 5). In addition, after evaluating these values with nurse management, additional values emerged: (an improvement in) quality of care, and interoperability or compatibility with systems that are currently in use.

The requirements resulting from the requirement analysis are classified into a) content, b) technical, and c) device (or implementation) requirements. Table 1 shows examples of participant quotes, attributes and resulting requirements.

**Table 1:** Example of quotes and requirements

**Quote**	**Attribute (“voice of the customer”)**	**Requirement**
“*but you would still have to gather it, search for it. In the end (…)”* (Pharmacist) *“(…) in one spot. All in one spot.”* (Nurse 1)	One stop portal for information	All information is accessible via one interface or starting point.
*“So I think you would really have to carry it around with you, but then the question is, how to carry it precisely.”* (Nurse 1) *“It should fit into a pocket.”* (Nurse 5)	Mobility of information	Size of device should be bigger than smartphone, but small enough to be carried around easily/fit in pockets.
*“It should be possible, in X-care there is a diagnosis for admission. It should be possible that that is linked, that when you click on the diagnosis, you get general information on pneumonia, just to give an example. But only general information: what it is, how it is treated.”* (Nurse 6)	One stop portal for information	The system offers protocols and general information on diseases.

Some example requirements are that the application should provide information from the patient file (medical history, diagnosis, received treatment, test results, etc.) and that it should provide all (antimicrobial related) information via one interface.

**Figure 2:**
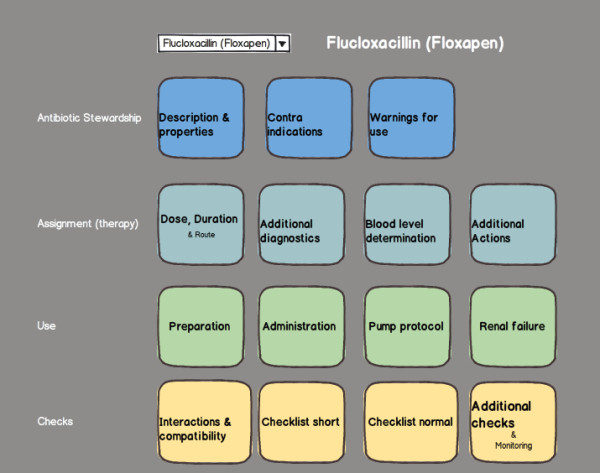
**Mock-up of information application dashboard.** In this mock-up, the general information was structured per antimicrobial in dashboard style.

For a complete overview of resulting requirements, we refer to Additional file 2. Based on the requirements, a mockup was created to roughly visualize a possible design and to communicate ideas to the design team and stakeholders. Figure 2 shows the mock-up of the principal screen where different types of information can be accessed.

### Observations

The observations indicate that several requirements do not optimally meet the two values: “ease of use” and “useful, relevant information” due to practical, organizational, or technical issues. For example, scanning the patient barcode appeared to be a user-unfriendly task, prone to work-around methods. Therefore, the requirement of disclosing patient information via a barcode scan lost its priority. Another issue was that nurses use a PC on wheels during drug administration. The application should therefore run on a PC as well as on mobile phones or tablets, as nurses prefer looking up information on a PC when they are already using that. However, as smartphones or tablets are easier to carry around, the application should function on those as well. Based on the observations, the design team decided that several requirements were dropped or postponed for the initial prototype. For example, disclosing patient-specific information and linking the concerning databases in one application is important, but this requirement was postponed because it is too costly and complicated for the prototype. This way, a prototype can be developed to create the necessary buy-in from the range of stakeholders (physicians, pharmacists, microbiologists, etc.) who are associated with the supply and maintenance of information, implementation, and technical support of the various information systems involved.

Based on the outcomes, a working prototype was developed that centralizes domain-specific antimicrobial-related information from different sources. It is web-based, enabling access via various devices (pc, tablet, smartphone) in different settings. After selecting the antimicrobial, the application displays different options for the types of task-supporting information in one single interface.

### Design

#### Card sorting task

Based on the card-sorts, an information structure for the application was identified. The sixteen-group solution as provided by the analysis program was deemed most applicable, regarding face validity of the groups (least illogical combinations). To prevent groups from being too small and to match closely related content, we merged some groups, which resulted in the following groups: dosage; preparation; administration; properties; acute responses and warnings; interactions, contra-indications, and compatibility with other drugs; and medical/for the physician. Item agreement percentages as they resulted from the card sort analyses are displayed in Table 2. On average, agreement on placing two items in one group ranges from 28% to 60%. This shows that some items were placed in groups that the participants did not agree on, and that some items were originally placed in isolated categories. This was especially the case in the ‘properties’ (28%) and ‘acute responses and warnings’ (39%) groups.

**Table 2:** Card sort item agreement per group

**Group**	**Number of cards**	**Average agreement (%) of two cards in this group**	**Example**
Dosage	2	60	Card Dosage source 1 and card Dosage source 2 were placed in the same group by 60% of the participants.
Preparation	7	57	Card “Pump protocol” and card “Final concentration of solution” were placed in the same group by 10% of the participants.
Administration	10	55	Card “Administration route” and card “Administration per perfusion” were placed in the same group by 30% of the participants.
Properties	5	28	Card “Extra checkups” and card “Blood tests” were placed in the same group by 80% of the participants.
Acute responses and Warnings	6	39	Card “Side effects” and card “Warnings and precautions” were placed in the same group by 40% of the participants.
Interactions, Contra-indications, Compatibility with other drugs	5	55	Card “Contra indications” and card “Interactions” were placed in the same group by 80% of the participants.
Medical/for the physician	7	60	Card “Kinetic properties” and card Available products” were placed in the same group by 50% of the participants.

Safety was added as a group based on participant comments after the card sort. Furthermore, ASP experts (clinical microbiologist and pharmacist) suggested additional content and, thus, additional groups. The groups were divided into five categories for the dashboard-style overview page: application, precautions/details, background information, safety, and medical information/other. Where groups had to be merged, original card-sorting group results were kept as subcategories (buttons in the overview page) as much as possible.

#### Information searching task and prototype evaluation

Based on the scenario-based searching tasks and prototype evaluation, various suggestions for prototype improvement and the implementation plan were identified (here, we focus on the variety of suggestions, not how often they came up). Table 3 gives an overview of the results.

**Table 3:** Re-design results based on scenario tests and prototype evaluation

**Suggestion description**	**Example quotes**	**Number of (unique) results**
Content: Remarks or events that indicate what information, and how much information should or should not be incorporated in the app. Remarks or events that indicate the usefulness or importance of certain content	*“Protective measures, is it already in the app? Or in a protocol? (…) For your own protection”* (Nurse 3)*. “We are talking about antibiotics, but would it be possible to use this for other medications as well?” (*Nurse 5).	41
Structure: Remarks or events that indicate how information should be structured or displayed	*“For example, the Farmacotherapeutisch Kompas [website with pharmaceutical information] is very nice, but too complicated. Difficult to understand, at a glance. And this [the app] is just, this is very nice at a glance”* (Nurse 10)*. “This is clear, everything I need is on this page”* (Nurse 2). *“Here for example, it is a lot of text. It would be better to make more subheadings or something”* (Nurse 5).	13
Layout: Remarks about the layout; what the app looks like	*“That it looks very good”* (Nurse 10).	5
Functionality: Remarks or events that indicate what the app should or should not be able to do. Remarks or events that indicate how it functions/should function	*“I would print pump protocols. I do that sometimes, I put them in the patient file (…) maybe it is me, if I would have a nice iPad, I would find that convenient as well”* (Nurse 3)*.*	13
Task perception: Remarks on, or events that describe current tasks or work methods, and remarks or events that indicate perception of what own task or physician tasks constitute	*“We can have a look at it, but we cannot do anything about it. In principle, the physician is responsible for lab results. Sometimes we alert the physician when we see it, but we do not pay attention to these things, with antibiotics”* (Nurse 1). *“It is no tour task, really, but I think, you all work with people for people, so (…) you should be able to do something about it (…) because we are the ones, normally, who administer it.”* (Nurse 6).	22
Implementation: Remarks or events that indicate how the app relates to the current information infrastructure or how it would fit into current work processes	*“I think this is something for the physician, whether it is a small-spectrum or broad-spectrum antibiotic (…) for us, preparation and how you should administer it is important”* (Nurse 2). “*We are doing a lot on the computer already, I think it would best to have everything in one place, that you are with the patient and [have the information at hand]. We are heading that way anyway”* (Nurse 9).	8

**Figure 3:**
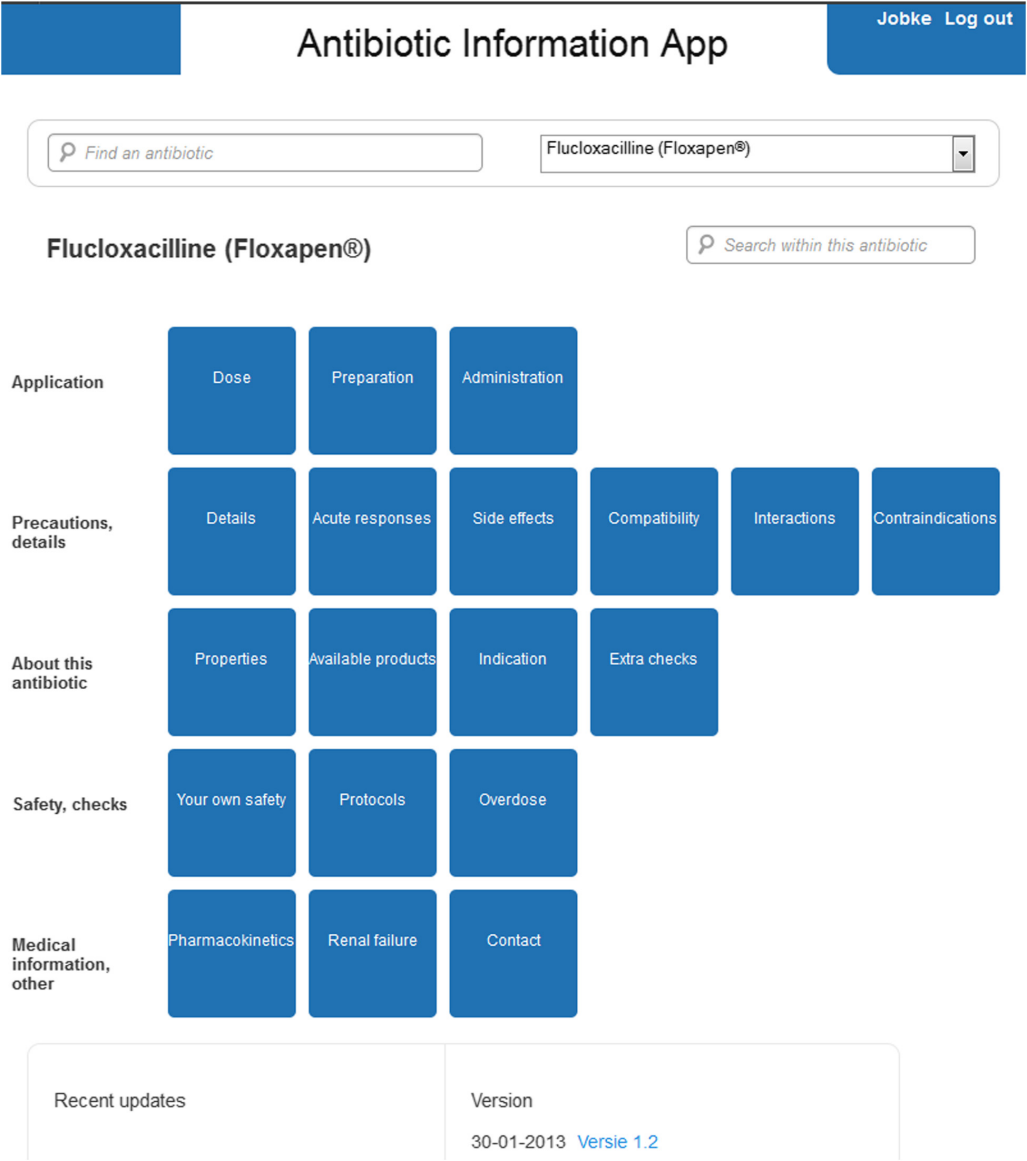
**Screenshot of information application.** The application consists of buttons, grouped into categories corresponding to the card sort results, with minor changes.

Besides appraisal and concrete suggestions for improvement regarding content, structure, functionality, layout and implementation, more fundamental remarks on task perception arose. The results show that the role perception of nursing in ASPs is undefined regarding the complex aspects of nurse tasks: monitoring of patient progress, checking for critical events, and alerting or alarming others in case of alarming changes in patient status.The information search task and prototype evaluation results were discussed with stakeholders to assess the feasibility from a medical, organizational, and technical perspective. This resulted in the rejection of some requirements, such as the expansion of the content to other types of medications (since that would render the project too large for this phase). The finalization of the application and concurring implementation strategy were adjusted to these outcomes and stakeholder discussions. For example, in instructional meetings extra attention was paid to task and role views of nurses in an ASP, because there was ambiguity on this issue. A screenshot of the final application as it is implemented and tested on efficacy can be seen in Figure 3.

## Discussion

In this study on nurse information support in ASPs, we identified and studied the main problem areas (scattered sources and task-supporting infrastructure), by participatory development with the target group (nurses) as well as other stakeholders. Weighing user needs against the limitations caused by the context and stakeholder needs guided decision-making in the development process. This broad view is likely to contribute to a successful implementation [17]. In this study, the use of different methods and continuous feedback about results to the participants gained understanding of work practice (needs, tasks) and elicited requirements for the technology. At the same time, other stakeholders, such as managers and technology experts, were consulted to prioritise and operationalise the requirements. Besides elicitation of and decisions on functional requirements (what the technology should be able to do) and design requirements (what interfaces should look like and how they should be structured to support tasks), the dynamic of our approach resulted in insight regarding information needs, task perception, and implementation.

Nurse involvement in ASPs relies on being able to correctly perform basic antimicrobial-related tasks such as preparing, administering, checking and monitoring of antimicrobial use. In addition recognizing moments for treatment optimization is crucial. In this study we identified the information needs of nurses to perform these tasks adequately, and the problems that they are currently facing: a multitude of scattered information sources that are non-supportive. This complex work context is also visible in more general nursing settings concerning information use for drug-related tasks [13,18,19]. Nurses often need to interrupt their activities to search for information. The extent to which nurses actually interrupt their activities to search is unclear, but our observations indicate that the search is likely to be postponed (or dismissed altogether), possibly compromising patient care. Therefore, a good understanding of (work) practice and related problems is a first step in eHealth development. Based on our contextual inquiry we argue that for the improved use and monitoring of antimicrobials by nurses, supportive technology needs to integrate information from different (expert-driven) sources in a user-centered, task-supporting way. Integration of different sources into one display alone probably does not suffice and restructuring or even re-creating (including wording and phrasing of) information sources to specifically match nurses’ tasks may be necessary.

If nurses are to adopt an active role in ASP, they need background information about the basic principles of antimicrobials and antimicrobial stewardship [4]. However, we first need to focus on information provision at a more fundamental level, namely the information and instructions that support the tasks of nurses working with antimicrobials. In line with our results there are many occasions during their day-to-day work when nurses need to have quick and easy access to information about drug therapy, as Cogdill demonstrated [18]. Optimizing the information flow regarding drug therapy is a crucial first step. In addition, research shows that nurses acquire information and knowledge about drug administration by looking it up in written sources, or by asking colleagues, and not via formal training [18,19]. Memorizing all instructions and background information on all types of medications would be impossible. During the course of our research stakeholders maintained that even though knowledge should be present, it could also be implicitly acquired. Thus, reliable and easily accessible information is crucial for nurses as it may be a more important source than previously acquired knowledge or training [19]. We showed how this (often expert-based) information can be matched to work practice by continuous involvement of participants (users and other stakeholders) during eHealth development.

The unexpected finding regarding nurse role perception leads us to conclude that our approach may be especially useful in development processes where new programs or work methods are introduced, instead of merely a new technology. Target group involvement during formative research, combined with stakeholder checks or evaluations, rendered important results; it opened a discussion on work methods, tasks, and responsibilities that influence the technology (e.g., ambiguity on what content should be aimed at nurses) and helped to adjust the technology to work practice.

We started this study with the idea that nurse support in ASPs relies on good information support regarding antibiotics, as our first explorations into ASP support tools indicated [10]. The scenario-based task results of the study described in this article showed that providing (easy accessible) information on (for example) optimal dosage or drug-drug interactions, does —in itself— not trigger the nurse to be more proactive in signalling problems in these areas, even though the nurses did acknowledge their signalling potential. To support these implicit roles, we plan further studies. We aim to capture expert and nurse opinions to shape and concretize these roles, and develop adequate (technology) support, consisting of checklists or decision aids as these are promising in other areas [20].

### Limitations

Although our results regarding information needs are comparable to findings in other studies, as described above, the results may not be fully generalizable to other wards or specialisms. Given the stakeholder-centered approach we applied, this is to some extent inevitable. To ensure a good fit between users, the organization, and the technology, one needs to actively involve stakeholders in the development process, and the qualitative and iterative nature of the methods that were used imply small research samples. However, small samples can produce valuable results when applying user-centered methods such as card sorts and scenario-based user tests [21,22], and we were able to perform multiple iterations in the development process. We tried to address issues of representativeness by involving participants with varying background and work experience in the research.

Another possible limitation of the study is a current lack of insight into uptake and effectiveness of the application on actual work practice. Even though the user tests did hint towards satisfaction with this way of information searching and representation, the effects of the application on work efficiency, medication errors, and changes in nurse performance regarding antimicrobial stewardship (increased awareness, alarming, more appropriate antimicrobial therapy) are unknown. Therefore, the release version of the application will be tested in a six-month pilot study that focuses on effects on information behavior, satisfaction, and nurse behavior in antimicrobial stewardship. Also, stakeholders are consulted to create business models for the application in order to determine an implementation strategy that reaches beyond the pilot ward.

## Conclusions

This article shows how participatory development can be applied to study and innovate health care. Via this approach, we found that nurses can be valuable contributors to ASPs, provided their information needs are met. The participatory development methods allowed us to explore this novel concept matching top-down (expert-based views, medical protocols) and bottom-up (nurses’ information needs) perspectives in a complex information context. The requirement elicitation methods and evaluations of the concept rendered results that have implications reaching beyond technology design: The methods support and shape health care innovation and, in this case, resulted in co-creation of technology, generated commitment, and aided antimicrobial stewardship by nurses.

## Competing interests

The authors declare that they have no competing interests.

## Authors’ contributions

JW was involved in all phases of the study. LvV was involved in the design, data analysis and drafting of the manuscript. MvL, NdJ, JK, RH and LvGP participated in the design, and coordination of the study, and helped to draft the manuscript. All authors read and approved the final manuscript.

## Pre-publication history

The pre-publication history for this paper can be accessed here:

http://www.biomedcentral.com/1472-6947/14/45/prepub

## Supplementary Material

Additional file 1 Nurses’ patient-specific antimicrobial information needs. Click here for file

Additional file 2 Information Application Requirements. Click here for file
