# Supplementary material for: Participatory eHealth development to support nurses in antimicrobial stewardship
Source: BMC Med Inform Decis Mak. 2014 Jun 5;14:45. doi: 10.1186/1472-6947-14-45 (PMC4074392; doi:10.1186/1472-6947-14-45)
Supplement: Additional file 1 — Nurses’ patient-specific antimicrobial information needs. [file 1472-6947-14-45-S1.doc]

Additional file 1 Nurses’ patient-specific antimicrobial information needs

| *Phase of care process* | *Patient-specific content* |
| --- | --- |
| Admission of patient | Who is this patient? (identity/background/general information) |
|  | Why was this patient admitted?  (diagnosis/reason for admission) |
|  | What medication/treatment has the patient already received at the emergency unit/other ward? |
|  | Has the patient been taking antimicrobials at home? With what effect? |
|  | What is the medical history of the patient, including allergies, earlier admissions? |
|  | What additional diagnoses/tests have already been performed? (cultures taken, X-rays) |
|  | What medication or treatment was prescribed for this patient and needs to be executed by the nurse? |
|  | Are test results already available? |
|  | What room/bed is assigned to the patient? |
| Preparing and administering medication | What medication/treatment is prescribed to this patient, including type, frequency, dose, administration method, duration? |
|  | What medication/treatment has the patient already received? |
|  | What is the weight of the patient? |
|  | How to act when patient has renal failure? |
|  | Is there a request to determine blood levels? |
| Monitoring the patient | Who is this patient? (identity/background/general information) |
|  | Why was the patient admitted? (diagnosis) |
|  | What allergies does the patient have? |
|  | What medication or treatment has been prescribed for this patient? |
|  | What is the condition of the patient? (general clinical assessment, temperature, blood pressure, pulse, fluid checks, and test results) |
|  | How to recognize medication side effects, or symptoms that indicate allergy present in this patient? |
|  | How to respond to impairments such as allergies, renal failure, fluid restriction? (e.g., by performing extra tests) |
| Discharging the patient | What aftercare is required, what aids should be in place for homecare? |
|  | What medication should the patient take at home, and how? |
|  | Are checkups required? |
|  | Are additional tests required? (lung function) |
|  | Have patient transport arrangements been made? |
