# Supplementary material for: Participatory eHealth development to support nurses in antimicrobial stewardship
Source: BMC Med Inform Decis Mak. 2014 Jun 5;14:45. doi: 10.1186/1472-6947-14-45 (PMC4074392; doi:10.1186/1472-6947-14-45)
Supplement: Additional file 2 — Information Application Requirements. [file 1472-6947-14-45-S2.doc]

Additional file 2 Information Application Requirements

| *Type* | Requirement |
| --- | --- |
| *Content* | The application should disclose information that is patient-specific:   - Prescribed treatment and medication - Information from the patient file: medical history, diagnosis, received treatment, test results, etc. - Special alerts/consideration points that need to be looked after   The application should disclose information that is domain-specific:   - Background information about antimicrobials, including guidelines and protocols for use; and - Background information about the disease or syndrome of a patient. |
| *Technical (layout, navigation and interactivity)* | All (antimicrobial-related) information is accessible via one interface;  The primary window of the application discloses content without any need to scroll down;  The application displays the available content in one overview (see Figure 6);  Information is linked—navigating through related information is possible by touching or clicking on a (hyperlinked) word;  Databases for patient-specific and general information are integrated;  The application discloses real-time patient information;  Patient information in the patient file can be modified or entered via the application; and  Patient-related information can be retrieved by scanning the patient’s barcode bracelet. |
| *medium (device)* | The application is mobile;  The size of the device should be bigger than a telephone, but the device should fit into a pocket/be carried around easily;  It must be possible to attach the device to a pocket/coat/clothing;  The device should be located near each patient’s bed;  Each nurse should be able to carry a device with him/her; and  One device should be available per (patient) room. |
